# Exploring the feasibility of establishing a core set of sexual, reproductive, maternal, newborn, child, and adolescent health indicators in humanitarian settings: results from a multi-methods assessment in Jordan

**DOI:** 10.1186/s12978-023-01589-w

**Published:** 2023-04-03

**Authors:** Majd Hammad, Angel M. Foster, Anya Aissaoui, Emily Clark, Kaeshan Elamurugan, Kanya Lakshmi Rajendra, Ieman Mona El Mowafi, Loulou Kobeissi

**Affiliations:** 1 Hakoura, Amman, Jordan; 2 Institute for Population Health, University of Ottawa, Ottawa, ON Canada; 3 Cambridge Reproductive Health Consultants, Cambridge, MA USA; 4 Faculty of Health Sciences, University of Ottawa, Ottawa, ON Canada; 5 NORImpact Consultancy AS, Rytterfaret 17A, Hafrsjord, Norway; 6 Department of Health Behavior and Health Education, School of Public Health, University of Michigan, Ann Arbor, MI USA; 7 Department of Sexual and Reproductive Health and Research (SRH), World Health Organization, Geneva, Switzerland

**Keywords:** Jordan, Monitoring and evaluating, Sexual and reproductive health, Maternal, child and adolescent health, Humanitarian data reporting, Health Information Systems

## Abstract

**Background:**

Reliable and rigorously collected sexual, reproductive, maternal, newborn, child, and adolescent health (SRMNCAH) data in humanitarian settings is often sparse and variable in quality across different humanitarian settings. To address this gap in data quality, the World Health Organization (WHO) developed a core set of indicators for monitoring and evaluating SRMNCAH services and outcomes in humanitarian settings, and assessed their feasibility in the field in Jordan, in addition to three other countries; with the goal of aggregating information from global consultations and field-level assessments to reach consensus on a set of core SRMNCAH indicators for services and outcome evaluation in humanitarian settings among WHO global partners.

**Methods:**

The feasibility assessment in Jordan focused on the following constructs: relevance/usefulness, feasibility of measurement, systems and resources, and ethical issues. The multi-methods assessment included five components; a desk review, key informant interviews, focus group discussions, facility assessments, and observational sessions.

**Results:**

Findings suggest that there is widespread support among regional, national, as well as global stakeholders for developing a core list of SRMNCAH indicators for monitoring and evaluation of services and outcomes in humanitarian settings in Jordan. There are numerous resources and data collection systems that could be leveraged, built upon, and improved to ensure the feasibility of collecting this proposed set of indicators. However, the data collection load requested from donors, the national government, international and UN agencies, coordination/cluster systems must be better harmonized, standardized, and less burdensome.

**Conclusions:**

Despite stakeholder support in developing a core set of indicators, this would only be useful if it has the buy-in from the international community. Greater harmonization and coordination, alongside increased resource allocation, would improve data collection efforts and allow stakeholders to meet indicators’ reporting requirements.

## Abstract (plainlanguagesummary)

In humanitarian settings, data collection is often unreliable and not standardized – especially with regards to sexual, reproductive, maternal, newborn, child, and adolescent health (SRMNCAH). In order to address this gap in data quality, the World Health Organization proposed a list of core indicators to four countries, including Jordan. The goal was to use the findings from the Jordanian context to contribute to the determination of a core set of indicators, and accumulate a list of necessary policy, funding, infrastructure, capacity and training that will be required. This study had two components: a feasibility assessment and a multi-methods assessment. The feasibility assessment in Jordan focused on the relevance of the proposed indicators, the feasibility of measurement, the resources in place, and ethical issues. The multi-methods assessment included a desk review, interviews with key informants and focus groups, and facility assessments (including observational sessions). The findings showed that partners in Jordan supported developing a standard set of SRMCAH indicators that can be used among all humanitarian partners in the country. There are a number of systems in place that could be used or strengthened to improve data collection. However, the findings also showed that there must be better collaboration and coordination between the different partners in Jordan, as well as increased resources, to alleviate burden on front-line staff, starting with standardizing and strengthening Jordan’s health information system (HIS). In conclusion, though there is a desire to harmonize indicators, more input and resources are needed from the international community to aid in the standardization of data collection in order to meet local reporting requirements and ease burden on local staff.

## Background

### Syrian crisis in Jordan

As the Syrian crisis continues, Jordan hosts more than 650,000 registered Syrian refugees in both urban and camp settings [1], and another estimated 1.2 million unregistered refugees in urban and rural areas across the country [2]. The majority of Syrian refugees in Jordan (79%) live outside refugee camps [1] in urban and rural areas throughout the country, and Syrians who reside in these communities have less access to, or have more difficulty navigating, local health services [3]. As of 2021, approximately fifty percent of refugees in Jordan are women and girls. Of this population, 160,000 are women of reproductive age [2], an estimated 16,000 of whom are pregnant at any given time [4]. More than half of these pregnant women are under the age of 25 [5]. This has caused considerable strain on Jordan’s already weak existing infrastructure and consequently imposed many implications on sexual, reproductive, maternal, newborn, child, and adolescent health (SRMNCAH) services, access, and outcomes [2, 3, 6, 7].

### SRMNCAH data collection and indicator reporting

Humanitarian emergencies are frequently characterized by the collapse and fragmentation of basic health services [8, 9]. In addition to the strain caused by humanitarian emergencies on host countries’ health systems and services, the large influx of refugees into Jordan has placed an unsustainable strain on the economy [10, 11], infrastructure, and security systems [12]; consequently, organizations are unable to meet the simultaneous demands of both refugees and its residents [9]. For better decision-making, coordination and response in crisis settings such as Jordan, multi-lateral and bilateral humanitarian agencies and their implementing partners need access to appropriate information [13–15]. Over the last few decades, researchers have extensively reported that humanitarian emergencies could generally lead to either a shortage or an overload of information—and both situations impair the provision of effective humanitarian assistance [15, 16].

Timely and rigorous collection, aggregation, and use of SRMNCAH data for services and outcomes evaluation in humanitarian settings is an important component of accountability and transparency within the aid community, and most importantly, accountability for the populations that the programs and developments are catered to [17]. Collecting complete, reliable, and quality data is also key to increase aid efficiently and effectively for populations affected by emergency and humanitarian crises [16, 18]. Transparency, accountability and measurement of the impact of humanitarian interventions are not only important for humanitarian organisations but are often requirements set by donors [15, 19]. Rigorous data collection and analysis would contribute to measurable assessments of the objectives, goals, and purpose of humanitarian initiatives in terms of process, impact, and intended outcomes [20, 21].

### SRMNCAH data collection and indicator reporting in Jordan

In the Jordanian context, multiple concerted efforts have been made to address the gaps in availability of SRMNCAH data. The most recent of such efforts is led by the Higher Population Council National Strategy for Reproductive Health and Family Planning (NSRHFP) by creating guidelines to support and engage key stakeholders in the coordination of all sexual and reproductive health (SRH) efforts in the country, including the data needed to meet the agreed upon national goals and global standards of care [22]. However, with a three-tiered health care system, limited budgets, and varying policies and regulations, the development of national priorities and guidelines for improving SRMNCAH indicators’ reporting for its population—and for refugee populations more specifically – has proven to be challenging [23].

### Aims and objectives

In light of the above, WHO, in close coordination with local, regional, and global partners, agreed to test the feasibility of a candidate set of SRMNCAH indicators for humanitarian settings at the field level. This was done in four countries experiencing different types of humanitarian crises, including Jordan, to determine the feasibility, relevance, and acceptability of these indicators. In the article, we discuss the results of this assessment in Jordan. The assessment took place in both camp and urban settings in the capital city of Amman as well as in Irbid and Mafraq (Al-Za’atari), and Karak and Aqaba in the North and South regions of Jordan, respectively.

By assessing feasibility, we aimed to explore the potential impact of the intended data collection and analysis, whether or not national and non-governmental monitoring and evaluation systems have the needed resources to collect SRMNCAH indicators, and the ability of the system to adhere to ethical practice and safeguard clients’ confidentiality and privacy. The results of Jordan’s country level assessment will be eventually aggregated with the results from other field-level assessments in order to reach a global consensus on a minimum set of core SRMNCAH indicators for services and outcomes evaluation in humanitarian settings among donor agencies, UN agencies, and international NGOs working in humanitarian settings. Specifically, this paper focuses on providing an overview of the assessment results in Jordan to determine the feasibility, relevance, and acceptability of the M and E framework, based on the perception of the multiple stakeholders of this assessment.

## Methods

### Study design

**Fig. 1:**
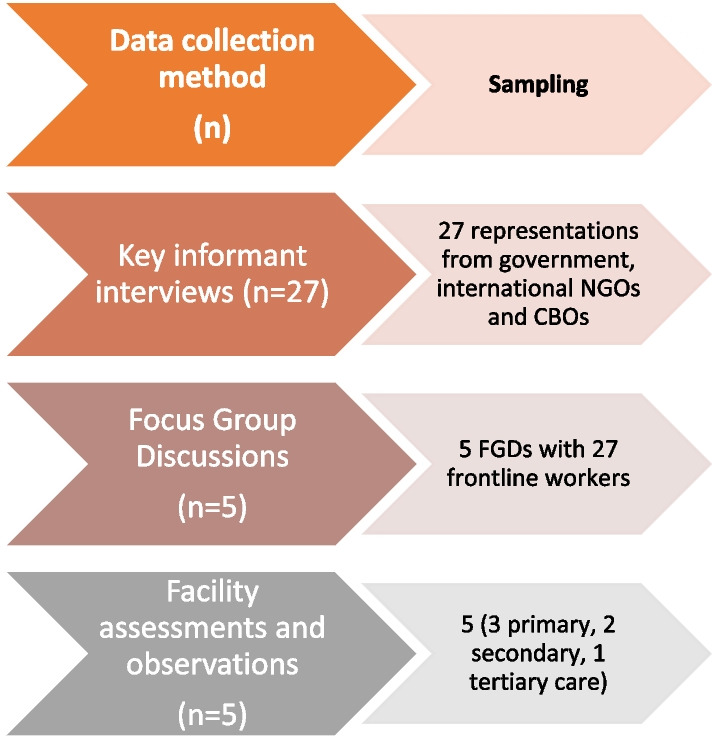
Data collection for Phase II. *NGO* Non-governmental organization; *CBO* Community based organization

This multi-methods assessment consisted of five main components: (1) a desk review of published articles and reports as well as internal documents (in English and Arabic); (2) key informant interviews with representatives from government entities, international non-governmental organizations, and community-based organizations; (3) facility assessments at primary, secondary, and tertiary facilities that provide services to refugees in Jordan; (4) observation sessions focused on the logistical, ethical, and privacy practices associated with data collection and storage at select facilities; and (5) focus group discussions (FGD) with frontline workers at primary, secondary, and tertiary health centers (see Fig. 1).

The assessment centered on seeking and understanding different stakeholders’ perceptions and attitudes towards: SRMNCAH issues in Jordan, SRMNCAH service provision in Jordan for refugee populations, current reporting practices on SRMNCAH indicators, and the feasibility of reporting on the candidate set of core SRMNCAH indicators; and also the necessary buy-in needed from the sector to successfully nationally scale up, endorse and report against these indicators.^1^

### Desk review

The project was initiated with a comprehensive review of peer-reviewed literature, existing published and unpublished data, including institutional and donor reports that focused on SRMNCAH indicators’ reporting and analysis in Jordan; coupled with an in-depth examination of the national SRMNCAH indicators’ list that organizations are required to report against. This desk review also helped informed the selection of the target populations for each of the KIIs and FGDs.

### Field-level assessment (See Fig. 1).

#### Key Informant Interviews (KIIs)

We compiled a list of key agencies working and providing SRMNCAH services to Syrian refugees across Jordan; as the majority of offices for these agencies were headquartered in Amman, we interviewed KIs individually or in small groups in Amman, Jordan. We conducted 10 KIIs with 27 representatives from different entities including government entities, and national and international non-governmental organizations between January and February 2020. Using a semi-structured interview guide developed specifically for the overarching study, we focused on KIs’ perceptions and attitudes towards: SRMNCAH issues in Jordan, SRMNCAH service provision in Jordan for refugee populations, current reporting practices on SRMNCAH indicators, and the feasibility of reporting on the candidate set of core SRHMNAH indicators. We also explored stakeholders’ perceptions and attitudes of current challenges in documenting and resources needed to successfully report against these indicators. We further explored the necessary buy-in needed among donor, governmental, and non-governmental agencies to enable the success of this effort.

#### Facility assessments

A list of facilities that provided RH services to Syrian refugees in Al-Za’atari camp, Irbid, and Karak was compiled by level (primary, secondary, and tertiary facilities). Each facility was informed, and WHO and United Nations Population Fund (UNFPA) country offices in Jordan facilitated the needed authorization prior to the evaluation. A total of five facility assessments were conducted. These assessments aimed to determine the nature and extent of SRMNCAH services offered, the ways in which patient information was collected, logged, stored, and safeguarded, and the types of human and technological resources used in data capture. In conjunction with the facility assessments, observational sessions were also carried out in all five facilities. These observational sessions aimed to assess existing resources currently being employed to collect data and additional resources needed to collect additional needed data for the core set of SRMNCAH indicators.

#### Focus group discussions

The facilities identified as the largest subsidized providers for SRMNCAH services for Syrian refugees in their respective governorates were selected for the FGDs. Five FGDs were conducted with 27 frontline workers from the same five agency health care clinics. Participants provided verbal consent at the beginning of each FGD, which lasted an average of 90 min and took place in Arabic. With consent, we audio-recorded all five FGDs, debriefed as a team after each discussion, and wrote analytic memos to capture group dynamics and identify early themes.

### Analytic approach

An iterative, multi-phased approach was employed to analyse the data [24, 25]. All KIIs and FGDs were analysed for content and themes, using both inductive and deductive techniques, which were then combined with results from the facility assessments, validation discussions with KIs, and feedback from the WHO led to the final recommendations. The analysis focused on the four core elements: (1) feasibility of collecting the proposed core set of SRMNCAH indicators, (2) relevance and usefulness of SRMNCAH data management mechanisms; (3) availability of existing resources and systems for national and humanitarian SRMNCAH data collection; and (4) ethical considerations of collecting and storing data.

### Research ethics

The Research Project Review Panel (RP2) of WHO’s Department of Sexual and Reproductive Health reviewed and approved this study. We also obtained authorization from the WHO’s country office in Jordan and the Jordanian Ministry of Health (MoH). The Social Sciences and Humanities Research Ethics Board of the University of Ottawa provided ethical approval (Protocol number: S-08-18-1029).

## Findings

The results of this assessment focused on the four core elements highlighted in the study objectives: (1) feasibility of collecting the proposed core set of SRMNCAH indicators, (2) relevance and usefulness of SRMNCAH data management mechanisms; (3) availability of existing resources and systems for national and humanitarian SRMNCAH data collection; and (4) ethical considerations. We first start by outlining the feasibility of the collecting the proposed SRMNCAH indicators, then we move to describing the current and potential advantages as well as challenges with SRMNCAH data capturing, followed by outlining the available data collections systems for the proposed indicators by the different humanitarian agencies. The findings section will conclude with discussing the different enforced measures in Jordan to protect data privacy and confidentiality among the different implementing agencies.

### Feasibility

The findings of this assessment indicated that 48% of the proposed indicators were considered relevant and feasible to collect (see Table 1); many of the STI and RTI (100%), newborn (81%), contraception (75%), maternal (53%), and abortion (40%) indicators were perceived to be relevant and feasible (see Tables 1 and 2). Of these indicators recommended for inclusion; 45% of indicators, reportedly, are currently being collected and 14% of those that are not currently being collected were perceived to be able to be collected given resources and trainings (see Table 2). The findings also indicated that 48% of the proposed indicators were deemed unfeasible or irrelevant; especially indicators in child (30%) and adolescent health (17%), HIV, and prevention from mother to child (PMTC) (Table 2).

**Table 1:** Summary and percentage of the indicators by domain that were perceived relevant and to the Jordanian context, by number and percentage respectively

	Number of indicators by domain (n)	Number of indicators relevant to the Jordanian context (n)	% of indicators that are feasible
Contraception	4	3	75
Comprehensive Abortion Care	5	2	40
Maternal Health	17	9	53
Newborn Health	16	13	81
Child Health	10	3	30
Adolescent health	6	1	17
Sexual and gender-based violence	7	3	14
HIV	3	0	0
Prevention from Mother to Child	4	0	0
Sexually transmitted infections (STIs) and reproductive tract infections (RTIs)	1	1	100
Total	73	35	48

**Table 2:** Summary findings of the feasibility of collecting the following proposed SRMNCAH indicators in the humanitarian context of Jordan

No	Indicator name	Overall % of agencies reporting	Place of collection	Facilitators to routine collection	Barriers to routine collection	Necessary modifications	Resources needed for routine collection	Exclude/include
Contraception								
1.1	Number of clients initiating contraception	60%	Health facilities, primary, and secondary health services	National Reporting System: centralized system in the MoH, NSSRH (Higher Population Council)	Insufficient security and privacy measures set in place	Clarification on the wording surrounding "initiating"—standardize definition of new user vs. recurrent user	Resources and training regarding security & data privacy	Include
1.2	Number of clients receiving emergency contraception	20%	Health facilities, primary, and secondary health services	National Reporting System: centralized system in the MoH, NSSRH (Higher Population Council)	No availability of commodity: No dedicated emergency contraceptive (EC) pill registered in Jordan Insufficient security and privacy measures set in place	N/A	Training on new IAFM guidelines Training providers on the different contraceptive modalities that can be used for EC Resources and training regarding security and data privacy	Include
1.3	Percentage of clients adopting modern contraceptive method after delivery	60%	Health facilities, primary, and secondary health services	National Reporting System: centralized system in the MoH, NSSRH (Higher Population Council)	Current collection occurring during PNC visit not at discharge Insufficient security and privacy measures set in place	Clarification on the wording surrounding "initiating"—standardize definition of new user vs. recurrent user	Resources and training regarding security & data privacy	Include
1.4	Percentage of clients adopting modern contraceptive method after abortion	0%	N/A	National Reporting System: centralized system in the MoH, NSSRH (Higher Population Council)	Legal status of abortion in Jordan Service not provided Insufficient security and privacy measures set in place	N/A	N/A	Exclude
Comprehensive abortion care								
2.1	Number of clients requesting an abortion	0%	N/A	N/A	Legal status of abortion in Jordan Potential risk for client and primary care provider Insufficient security and privacy measures set in place	N/A	N/A	Exclude
2.2	Number of clients receiving an abortion referral	30%		N/A	Legal status of abortion in Jordan Policy and legislative changes needed Insufficient security and privacy measures set in place	N/A	Training on the new IAFM guidelines Integration into HIS/existing data collection systems VCAT sessions Resources and training regarding security & data privacy Service mapping exercise at the health and/or humanitarian sector level to identify service provision of abortions	Exclude
2.3	Number of clients receiving an induced abortion	10%		N/A	Legal status of abortion in Jordan Policy and legislative changes needed Insufficient security and privacy measures set in place	N/A	Training on the new IAFM guidelines Integration into HIS/existing data collection systems VCAT sessions Resources and training regarding security & data privacy Service mapping exercise at the health and/or humanitarian sector level to identify service provision of abortions	Exclude
2.4	Number of clients presenting for post-abortion care (PAC)	40%		N/A	Legal status of abortion in Jordan Policy and legislative changes needed Insufficient security and privacy measures set in place	N/A	Training on the new IAFM guidelines Integration into HIS/existing data collection systems VCAT sessions Resources and training regarding security & data privacy Service mapping exercise at the health and/or humanitarian sector level to identify service provision of PAC	Include
2.5	Number of clients receiving PAC	100%		N/A	Legal status of abortion in Jordan Policy and legislative changes needed Insufficient security and privacy measures set in place	N/A	Integration into HIS/existing data collection systems Resources and training regarding security & data privacy Service mapping exercise at the health and/or humanitarian sector level to identify service provision of PAC	Include
Maternal health								
3.1	Number of maternal deaths	100%	Jordan Civil Registration and Vital Statistics System National reporting system utilized (MMSRS) with country-wide buy-in Resource and systems are centralized through MoH		N/A	N/A	N/A	Include
3.2	Number of maternal deaths, disaggregated	100%		Jordan Civil Registration and Vital Statistics System National reporting system utilized (MMSRS) with country-wide buy-in Resource and systems are centralized through MoH	N/A	Should include 15 and under category	N/A	Include
3.3	Percentage of maternal death reviews	100%	Secondary and tertiary facilities only	Jordan Civil Registration and Vital Statistics System National reporting system utilized (MMSRS) with country-wide buy-in Resource and systems are centralized through MoH	Applicable in secondary and tertiary facilities only	N/A	N/A	Include
3.4	Number of clients receiving antenatal care (ANC)	60%		N/A	Insufficient security and privacy measures set in place	N/A	Resources and training regarding security & data privacy	Include
3.5	Number of deliveries	70%		Jordan Civil Registration and Vital Statistics System	Birth registry not centralized in facilities Policy and legislative changes surrounding birth registry and reporting requirements of stillbirths Insufficient security and privacy measures set in place	N/A	Training on how to report stillbirths Centralizing birth registry in secondary and tertiary facilities Integration into HIS/existing data collection systems Resources and training regarding security & data privacy	Include
3.6	Number of deliveries, disaggregated	60%		Jordan Civil Registration and Vital Statistics System	Birth registry not centralized in facilities Policy and legislative changes surrounding birth registry and reporting requirements of stillbirths Insufficient security and privacy measures set in place	N/A	Training on how to report stillbirths Centralizing birth registry in secondary and tertiary facilities Integration into HIS/existing data collection systems Resources and training regarding security & data privacy	Include
3.7	Number of clients receiving post-natal care (PNC)	50%	Secondary and tertiary facilities only	N/A	Applicable in secondary and tertiary facilities only Insufficient security and privacy measures set in place	N/A	Resources and training regarding security & data privacy	Include
3.8	Number of caesarean section deliveries	60%	Tertiary facilities only	N/A	Applicable in tertiary facilities only Insufficient security and privacy measures set in place	N/A	Resources and training regarding security & data privacy	Include
3.9	Availability of PAC	0%	N/A	N/A	N/A	N/A	Service mapping exercise at the health and/or humanitarian sector level to identify service provision of PAC	Exclude
3.10	Availability of basic emergency obstetric care (BEmOC)	0%	N/A	N/A	N/A	N/A	Service mapping exercise at the health and/or humanitarian sector level to identify service provision of BEmOC	Exclude
3.11	Availability of comprehensive emergency obstetric care (CEmOC)	0%	N/A	N/A	N/A	N/A	Service mapping exercise at the health and/or humanitarian sector level to identify service provision of CEmOC	Exclude
3.12	Availability of skilled personnel	0%	N/A	N/A	Irrelevant to context as high quality medical care is readily available	N/A	N/A	Exclude
3.13	Number of antenatal care clients with tetanus vaccination	70%		N/A	Low burden of disease, service not routinely provided	N/A	N/A	Exclude
3.14	Number of ANC clients receiving preventive therapy for malaria	0%	N/A	N/A	Low burden of disease, service not routinely provided	N/A	N/A	Exclude
3.15	Number of ANC clients receiving syphilis screening	0%	N/A	N/A	Low burden of disease, service not routinely provided	N/A	N/A	Exclude
3.16	Number of ANC clients receiving urinary tract infection screening or treatment	0%	N/A	N/A	Low burden of disease, information not actionable	N/A	N/A	Exclude
3.17	Number of clients with identified maternal morbidities during post-natal care (PNC)	60%		N/A	Insufficient security and privacy measures set in place	Specify morbidity types	Resources needed to specify morbidity types (i.e.: training of staff and creating and distributing manuals on types of maternal morbidities during PNC) Resources and training regarding security & data privacy	Include
Newborn health								
4.1	Number of neonatal deaths	60%		Jordan Civil Registration and Vital Statistics System	No national system to review newborn death Insufficient security and privacy measures set in place	N/A	Resources needed to establish national systems to capture newborn death and review these cases for cause of death (development and implementation of a perinatal surveillance system) Training of primary care providers on how to report on neonatal death Resources and training regarding security & data privacy	Include
4.2	Number of stillbirths	60%		Jordan Civil Registration and Vital Statistics System	N/A	N/A	Training on how to report on stillbirths Integration into HIS/existing data collection systems	Include
4.3	Number of babies born low birth weight	60%		N/A	N/A	Specification of the weight (e.g.: < 2500)	N/A	Include
4.4	Number of small and sick newborns receiving care	60%		N/A	N/A	N/A	N/A	Include
4.5	Number of newborns receiving post-natal care	60%		N/A	N/A	N/A	N/A	Include
4.6	Availability of KMC	0%	N/A	N/A	Service not routinely provided; information not actionable	N/A	N/A	Exclude
4.7	Availability of neonatal resuscitation	0%	N/A	N/A	Irrelevant to context as high quality medical care is readily available In-patient health care system	N/A	Service mapping exercise at the health and/or humanitarian sector level to identify service provision of neonatal resuscitation	Exclude
4.8	Number of neonatal deaths, disaggregated	60%		Jordan Civil Registration and Vital Statistics System	No national system to review newborn death	N/A	Resources needed to establish national systems to capture newborn death and review these cases for cause of death (development and implementation of a perinatal surveillance system) Training of primary care providers	Include
4.9	Percentage of perinatal death reviews	10%		N/A	No national system to review newborn death	N/A	Resources needed to establish national systems to capture newborn death and review these cases for cause of death (development and implementation of a perinatal surveillance system) Training of primary care providers	Include
4.10	Number of newborns receiving Hepatitis B vaccine	0%	N/A	N/A		N/A	Integration into HIS/existing data collection systems	Include
4.11	Number of newborns initiating breastfeeding early	0%	N/A	N/A	Information not actionable	N/A	N/A	Exclude
4.12	Number of infants weighed at birth	0%	N/A	N/A	N/A	N/A	Integration into HIS/existing data collection systems	Include
4.13	Number of babies registered	0%	N/A	Jordan Civil Registration and Vital Statistics System	Civil registry not centralized in facilities		Resources needed to integrate civil registry in facilities	Include
4.14	Number of newborns receiving treatment for possible severe bacterial infection (PSBI)	0%	N/A	N/A	N/A	N/A	Integration into HIS/existing data collection systems	Include
4.15	Number of newborns admitted	60%	Secondary and tertiary facilities only	N/A	Applicable in secondary and tertiary facilities only	N/A	N/A	Include
4.16	Number of newborns with morbidities identified during PNC	0%	N/A	N/A	N/A	Specify morbidity types	Resources needed to specify morbidity types	Include
Child health								
5.1	Number of deaths of children under 5	0%	N/A	Jordan Civil Registration and Vital Statistics System	N/A	N/A	N/A	Include
5.2	Under 5 mortality rate	0%	N/A	N/A	Population level indicator with impractical denominator	Convert to facility-based indicator for routine collection	N/A	Exclude
5.3	Percentage of children under 5 with suspected pneumonia taken to appropriate health facility	0%	N/A	N/A	Population level indicator with impractical denominator	Convert to facility-based indicator for routine collection	N/A	Exclude
5.4	Coverage of diarrhea treatment	0%	N/A	N/A	N/A	N/A	N/A	Exclude
5.5	Percentage of children under 5 who are wasted	0%	N/A	N/A	Low burden of disease Population-level indicator with impractical denominator	Convert to facility-based indicator for routine collection	N/A	Exclude
5.6	Percentage of children under 5 who are registered	0%	N/A	Jordan Civil Registration and Vital Statistics System	Complicated by national registry system; system allows for a 30-day grace period for the male guardian to register the birth of the child which can result in lack of registration if a newborn death occurs within 30 days	Convert to facility-based indicator for routine collection	N/A	Include
5.7	Number of children presenting with fever tested for malaria in endemic settings	0%	N/A	N/A	Low burden of disease, service not routinely provided Specific infectious disease reporting requirements and management protocols for individual cases	N/A	N/A	Exclude
5.8	Number of confirmed cases of malaria in endemic settings	0%	N/A	N/A	Low burden of disease, service not routinely provided Specific infectious disease reporting requirements and management protocols for individual cases	N/A	N/A	Exclude
5.9	Percentage of confirmed malaria cases treated	0%	N/A	N/A	Low burden of disease, service not routinely provided Specific infectious disease reporting requirements and management protocols for individual cases	N/A	N/A	Exclude
5.10	Coverage of DP3	0%	N/A	N/A	Population- level indicator with impractical denominator	Convert to facility-based indicator for routine collection	N/A	Exclude
Adolescent health								
6.1	Adolescent birth rate	0%	N/A	National Reporting System: NSSRH (Higher Population Council)	Population-level indicator with impractical denominator	Convert to facility-based indicator for routine collection	N/A	Exclude
6.2	Sexual violence against children	0%	N/A	N/A	Information not actionable Population-level indicator with impractical denominator	Convert to facility-based indicator for routine collection	N/A	Exclude
6.3	Adolescent mortality rate	0%	N/A	N/A	Population-level indicator with impractical denominator	Convert to facility-based indicator for routine collection	N/A	Include
6.4	Percentage of adolescents living with HIV who are currently receiving antiretroviral therapy, disaggregated	0%	N/A	N/A	National HIV reporting requirements and management protocols for individual cases Population-level indicator with impractical denominator	Convert to facility-based indicator for routine collection	N/A	Exclude
6.5	Immunization coverage rate	0%	N/A	N/A	Population-level indicator with impractical denominator	Convert to facility-based indicator for routine collection	N/A	Exclude
6.6	Suicide rate, disaggregated	0%	N/A	N/A	Information not actionable Population-level indicator with impractical denominator	Convert to facility-based indicator for routine collection	N/A	Exclude
Sexual and gender-based violence								
7.1	Number of rape survivors	80%		National Information Management System for GBV	N/A	N/A	N/A	Include
7.2	Percentage of health facilities with clinical management of rape services	0%	N/A	National Information Management System for GBV		N/A	Service mapping exercise at the health and/or humanitarian sector level to identify service provision of clinical management of rape	Exclude
7.3	Percentage of rape survivors receiving HIV post-exposure prophylaxis	70%		National Information Management System for GBV	N/A	N/A	N/A	Include
7.4	Percentage of rape survivors receiving emergency contraception	30%		National Information Management System for GBV	No availability of commodity: No dedicated emergency contraceptive (EC) pill registered in Jordan Insufficient security and privacy measures set in place	N/A	Training on the new IAFM guidelines Training providers on the different contraceptive modalities that can be used for EC Resources and training regarding security & data privacy	Include
7.5	Number of rape survivors requesting abortion	0%	N/A	National Information Management System for GBV	Legal status of abortion in Jordan Potential risk for client and primary care provider Insufficient security and privacy measures set in place Service not routinely provided	N/A	N/A	Exclude
7.6	Number of rape survivors receiving induced abortion care or referral	0%	N/A	National Information Management System for GBV	Legal status of abortion in Jordan Policy and legislative changes Insufficient security and privacy measures set in place	N/A	Training on the new IAFM guidelines Integration into HIS/existing data collection system VCAT sessions Service mapping exercise at the health and/or humanitarian sector level to identify service provision of abortions Resources and training regarding security & data privacy	Include
7.7	Availability of intimate partner violence front line support (LIVES)	0%	N/A	National Information Management System for GBV	Service not routinely provided	N/A	N/A	Exclude
HIV								
8.1	Antiretroviral therapy coverage among people living with HIV, disaggregated	0%	N/A	National Reporting System controlled by the MoH	National HIV reporting requirements and management protocols for individual cases	N/A	N/A	Exclude
8.2	Percentage of exposed individuals receiving post-exposure prophylaxis	0%	N/A	National Reporting System controlled by the MoH	National HIV reporting requirements and management protocols for individual cases	N/A	N/A	Exclude
8.3	Percentage of donated blood units screened for HIV in quality assured manner	0%	N/A	National Reporting System controlled by the MoH	National HIV reporting requirements and management protocols for individual cases Not relevant outside hospital settings	N/A	N/A	Exclude
Prevention of mother-to-child transmission								
9.1	Percentage of antenatal care clients receiving syphilis screening and treatment	0%	N/A	N/A	National HIV reporting requirements and management protocols for individual cases	N/A	N/A	Exclude
9.2	Percentage of antenatal care clients offered testing for HIV	0%	N/A	N/A	National HIV reporting requirements and management protocols for individual cases	N/A	N/A	Exclude
9.3	Percentage of HIV-positive pregnant people receiving antiretroviral therapy	0%	N/A	N/A	National HIV reporting requirements and management protocols for individual cases	N/A	N/A	Exclude
9.4	Percentage of all deliveries to HIV-positive mothers receiving antiretrovirals	0%	N/A	N/A	National HIV reporting requirements and management protocols for individual cases	N/A	N/A	Exclude
Sexually transmitted infections (STIs) & reproductive tract infections (RTIs)								
10.1	Percentage of STI/RTI cases managed	70%		National Reporting System: centralized system in the MoH, NSSRH (Higher Population Council)	Insufficient security and privacy measures set in place	Include indicator on referrals to MoH	Develop materials and training on a wide range of STIs Training for healthcare providers Resources and training regarding security & data privacy	Include

The findings from this assessment revealed that Jordan has multiple existing data collection information systems. These systems often compete with each other, which results in gaps in data collection and place undue burden on frontline staff. Lack of internal capacity and staffing impede the ability of organizations to develop and implement comprehensive computerized data capturing and reporting systems across health facilities. Frontline workers are required to report to multiple systems utilizing various templates and requiring different information. With this pre-existing arduous process in place, frontline workers expressed reticence at collecting a list of this length and perceived that the standardization of indicators is necessary to achieve feasibility of their data collection. While many perceived that some of the new proposed indicators in the framework could be easily integrated into existing systems for collection; the feasibility of collecting an indicator did not correlate with the relevance of the indicator, as participants highlighted that such data collection necessitates the availability of the needed resources to ensure the accuracy of the collected data. In Table 2, we provide an overview of the included and excluded list of indicators, the reported percentage of agencies who are currently collecting these indicators; sites of data collection; perceived facilitators and barriers for routine data collection; and any necessary modifications and resources necessary for routine data collection.

The results showed that three of the four proposed indicators on contraceptives were feasible to collect (indicators 1.1–3) given some wording modifications. The indicator connected to post abortion care (PAC) (indicator 1.4) was suggested for exclusion due to the legal status of abortion care in Jordan. It was indicated that policy and legislative changes are needed in parallel with training on the legal status and new IAFM guidelines along with VCAT sessions for frontline workers to make abortion indicators feasible for data collection in the Jordanian context.

The maternal health indicators were perceived as fairly feasible to collect, with the exception of indicators on service availability in the maternal health sector (indicators 3.9-12). Participants perceived that these indicators are not feasible to collect because of low burden of disease and sporadic service provision (indicators 3.13-16). Most of the proposed newborn health indicators relied on facility-based information and therefore are either being collected or were perceived to be easily feasible to be collected (indicators 4.1–8). Participants, however, expressed concerns surrounding the way that neonatal deaths and stillbirth are defined, recorded, and audited, particularly for cases outside of camps (indicators 4.1-2). On the other hand, participants expressed concerns over the feasibility of collecting a number of indicators related to child (indicators 5.2-6) and adolescent health (indicators 6.2-6), because these proposed indicators are population-level indicators and participants were concerned with impracticalities associated with measuring the needed denominators and/or the low burden of disease (indicators 5.7–9) in the local context.

Our findings suggested that significant gaps exist around data collection for sexual and gender-based violence (SGBV)-related indicators (indicators 7.2-7). Deep concerns were expressed that public reporting of SGBV information could result in a de-prioritization of efforts to expand clinical management of rape and GBV services. While none of the agencies that participated in this project are currently reporting any data on pregnancy outcomes for rape survivors or related abortion requests (indicator 7.5), participants felt this information could be collected if appropriate resources, ownership, supports and training were adequately set in place. Finally, participants raised concerns about the inclusion of any of the HIV/AIDS-related indicators (indicators 8.1-3 and 9.1-9.4) due to major stigma and discrimination from service providers, and the legislative barriers to providing care to refugees in Jordan who are screened positively with HIV/AIDs; in parallel with the low prevalence of disease in Jordan. We provide additional information about the specific indicators proposed for each topic in Table 2 and provide a detailed narrative for each domain of indicators in WHO’s Jordan country-level report (available upon request).

### Relevance and usefulness of humanitarian SRMNCAH data management mechanisms

#### Perceived advantages with current and proposed SRMNCAH indicator reporting

All the different participants in this assessment agreed that accurate and reliable SRMNCAH data provide the opportunity for implementing evidence-based programming, defining priorities, ensuring accountability among implementors. Hence, the majority of the proposed indicators were perceived to be essential in order to improve the health outcomes for both refugee and vulnerable communities in Jordan. In addition to the benefits of meeting the needs of refugee and vulnerable populations, our KIs noted that the collection and reporting of SRMNCAH indicators allows organizations to monitor and evaluate whether SRMNCAH services provided meet global and regional standards of care, population health goals, and donor reporting requirements. As noted by one KI, “We can’t capture everything, we have to be strategic with which indicators we select. We prioritize of course the indicators that contribute to our country program documents, the ones that align with the Sustainable Development Goals and of course to meet our reporting requirements [to donors].”

However, stakeholders stressed the need for a core list of SRMNCAH indicators and encouraged that this same list to be used across all the different stakeholders simultaneously i.e. donors, the MoH, UN agencies, and international NGOs. In general, the stakeholders in all agencies supported the contours of the proposed core set of SRMNCAH indicators and noted that many of the specific indicators are aligned with many of their current and ongoing data collection practices and priorities. Stakeholders noted that across the board, that indicators derived from facility-level data were more relevant, useful, and practical to collect compared to those indicators that required information at the population level. As noted by an FGD participant, “The facility level indicators have both the potential to be more precise and really useful at the programmatic level in a way that population-level indicators tend to be harder to collect and are not necessarily accurate.” Stakeholders also repeatedly asserted that having a standardized set for SRMNCAH indicators is important for advocacy-related activities, including establishing or amending policies to help increase access to services for refugee populations and supporting requests for increased funding.

#### Perceived disadvantages with current SRMNCAH indicator reporting

Our findings also indicated several disadvantages that could be associated with collecting some of the proposed SRMNCAH indicators. As noted by many of our stakeholders and FGD participants, a multitude of barriers contribute to the significant under reporting for SGBV cases in Jordan. For instance, many of the stakeholders felt that publishing the low numbers of rape and other reported SGBV-related cases would undermine the prioritization of those services, as this would be interpreted as lack of demand for this service. As one KI explained, “Recording the data [SGBV cases] is okay but presenting it as an indicator that may be published to external agencies? These small numbers can be misleading. They will argue you don’t have a decent issue.” Other indicators equally perceived as controversial were some of those related to the adolescent health, child health and HIV/AIDs.

### Perceived gaps in the proposed SRMNCAH indicators

#### Indicators that should be removed from the core set of SRMNCAH indicators

Participants identified a number of the proposed indicators to either be not relevant or useful in the Jordanian context and therefore recommended their removal. The rationale for suggesting their exclusion, as per the KIs, revolved around one or more of the following: (1) the indicator relates to a condition for which there is low burden of disease in the Jordanian context; (2) national regulations and protocols restrict collecting information on the subject; (3) services related to the indicator are not (routinely) available; and/or (4) the indicator would not have any practical or actionable applications. Stakeholders were uniform in their belief that population-level indicators were not practical and could only be collected in conjunction with larger nationally representative surveys that take place every 5–10 years. More information on these specific indicators suggested for removal along with the reasons for removal can be found above in Table 3.

**Table 3:** List of indicators that should be excluded according to our stakeholders, with primary rationale

	Indicator number and name	Rationale for exclusion
Contraception	1.4: Percentage of clients adopting modern contraception method after abortion	• Service not provided • Insufficient security and privacy measures set in place
Comprehensive abortion care	2.1: Number of clients requesting an abortion	• Potential risk for the client and primary care provider • Insufficient security and privacy measures set in place
Maternal Health	3.9: Availability of PAC	• Service mapping exercise^a^ at the health and/or humanitarian sector level
	3.10: Availability of basic emergency obstetric care (BEmOC)	• Service mapping exercise at the health and/or humanitarian sector level
	3.11: Availability of comprehensive emergency obstetric care (CEmOC)	• Service mapping exercise at the health and/or humanitarian sector level
	3.12: Availability of skilled personal	• High quality medical care • In-patient health care system • Service mapping exercise
	3.13: Number of ANC clients with tetanus vaccination	• Low burden of disease, service not routinely provided
	3.14: Number of ANC clients receiving preventive therapy for malaria	• Low burden of disease, service not routinely provided
	3.15: Number of ANC clients receiving syphilis screening	• Low burden of disease, service not routinely provided
	3.16: Number of ANC clients receiving urinary tract infection screening or treatment	• Low burden of disease, information not actionable
Newborn Health	4.6: Availability of KMC	• Service not routinely provided; information not actionable
	4.7: Availability of neonatal resuscitation	• High quality medical care • In-patient health care system • Service mapping exercise
	4.11: Number of newborns initiating breastfeeding early	• Information not actionable
Child health	5.2: Under 5 mortality rate	• Population-level indicator with impractical denominator
	5.3: Percentage of children under 5 with suspected pneumonia taken to appropriate health facility	• Population-level indicator with impractical denominator
	5.5: Percentage of children under 5 who are wasted	• Low burden of disease, • Population-level indicator with impractical denominator
	5.7: Number of children presenting with fever tested for malaria in endemic settings	• Low burden of disease, specific infectious disease reporting requirements and management protocols for individual cases
	5.8: Number of confirmed cases of malaria in endemic settings	• Low burden of disease, specific infectious disease reporting requirements and management protocols for individual cases
	5.9: Percentage of confirmed malaria cases treated	• Low burden of disease, specific infectious disease reporting requirements and management protocols for individual cases
	5.10: Coverage of DPT3	• Population-level indicator with impractical denominator
Adolescent health	6.1: Adolescent birth rate	• Population-level indicator with impractical denominator
	6.2: Sexual violence against children	• Information not actionable, population-level indicator with impractical denominator
	6.3: Adolescent mortality rate	• Population-level indicator with impractical denominator
	6.4: Percentage of adolescents living with HIV who are currently receiving antiretroviral therapy, disaggregated	• National HIV reporting requirements and management protocols for individual cases, • Population-level indicator with impractical denominator
	6.5: Immunization coverage rate	• Population-level indicator with impractical denominator
	6.6: Suicide rate, disaggregated	• Information not actionable, • Population-level indicator with impractical denominator
Sexual and gender-based violence	7.2: Percentage of health facilities with clinical management of rape services	• Service mapping exercise at the health and/or humanitarian sector level
	7.5: Number of rape survivors requesting an abortion	• Potential risk for the client • Insufficient security and privacy measures set in place • Service not provided
	7.7: Availability of intimate partner violence front line support (LIVES)	• Service not routinely provided
HIV	8.1: Antiretroviral therapy coverage among people living with HIV, disaggregated	• National HIV reporting requirements and management protocols for individual cases
	8.2: Percentage of exposed individuals receiving post-exposure prophylaxis	• National HIV reporting requirements and management protocols for individual cases

^a^Given that Jordan does not have a nationally endorsed HIS, the indicators that ask for number of facilities, would either need to be reworded or conducted as a service mapping exercise at the health and humanitarian sector level. Service mapping exercise aims to examine what services and programs, and by whom are offered; the services and programs that other agencies within the same communities are offering. It seems to also examine the links between services and programs provided and utilized by the target population. Service mapping also aims to identify any service and/or programming gaps that may exist in the community that might need to be addressed [47]

All Jordanian stakeholders raised concerns about the inclusion of HIV/AIDS-related indicators. Our KIs explained that due to national regulations and policies, all HIV/AIDS related cases must be referred to the MoH and foreigners who test positive for HIV are subject to immediate deportation—including refugees. Other indicators were deemed irrelevant because services are not routinely provided in Jordan’s context, and policies and regulations hindering adequate capturing of data. These included indicators related to: comprehensive abortion care (CAC), syphilis screening, kangaroo mother care (KMC), and the LIVES^2^ intervention in cases of intimate partner violence. Indicators related to adolescent suicide rates and sexual violence against children were generally perceived as controversial for the participants of this study. KIs expressed that the indicator on suicide rate wouldn’t be actionable and presents as an outlier from the core list of SRMNCAH indicators. The findings also suggest that indicators with population-level denominators should be removed and even if it could be collected, the data wouldn’t inform programming due to, as it stands in Jordan, available population level data are unreliable.

#### Additional Indicators that should be added to the core set of SRMNCAH indicators

**Fig. 2:**
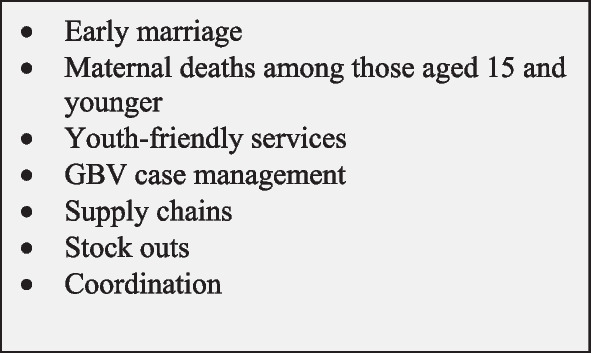
Topics that should be included in the core list of SRMNCAH indicators

Stakeholders in Jordan also proposed some additional indicators for inclusion to the core SRMNCAH list. Those suggested indicators were mostly focused on adolescent sexual and reproductive health, supply-chains, commodities/stock outs, and coordination. Related to the issue of child/early marriage, both KIs and FGD participants felt that the proposed maternal death indicator needs to be further disaggregated by age, as the risk of maternal morbidity and mortality differed for those 15 years of age and under compared to those aged 16–18. On Fig. 2, we provide a list of additional topics that the Jordanian stakeholders perceived should be included on any core SRMNCAH indicator list.

### Existing systems and resources for collecting SRMNCAH indicators

The findings from this assessment indicated that several robust health information systems are developed, implemented, and utilized by both international and national partner organizations in Jordan. In the last few years, there has been a significant national investment in the amelioration of national reporting systems for a subset of the proposed SRMNCAH domains, including systems for contraception, maternal health, and SGBV. Access to comprehensive, user-friendly, computerized systems, necessary staff, and capacity varied across agencies. As a result, agencies often had to input and analyze their data manually, impacting accurate, standardized, and timely data reporting. Although, a number of organizations have built their own dedicated HIS internally, not all agencies have access to the same level of funding and/or the same human and technological capacities, meaning that some agencies are working with less contextually developed systems. We provide a list of the available data collection resources and systems reported by our stakeholders in Table 4.

**Table 4:** Existing monitoring and evaluations systems reported by our stakeholders, by domain of indicators

SRNMCAH domain	Existing system
Contraception	• WizMonitor^a^ • Continuity of care workers^b^ • Internal HIS^c^ • Hakeem/manual • WizMonitor • Internal HIS
Comprehensive abortion care	• Continuity of care worker • Internal HIS • Patient charts • Miscarriage spreadsheet
Maternal health	• Resource and systems are centralized through the MOH
Newborn health	• WizMonitor • Continuity of care workers • Internal HIS • WizMonitor • Tally sheets/Internal HIS • Internal HIS
Child health	• WizMonitor • Continuity of care workers • Internal HIS • WizMonitor • Tally sheets/Internal HIS • Internal HIS
Adolescent health	• WizMonitor • Continuity of care workers • Internal HIS • WizMonitor • Tally sheets/Internal HIS • Internal HIS
Sexual and gender-based violence (SGBV)	• WizMonitor • K.N.A.C.K • Continuity of care workers • Internal HIS • WizMonitor • Tally sheets • Internal HIS
HIV	• System controlled by the MOH
PMTCT	• N/A
Sexually transmitted infections (STIs) and reproductive tract infections (RTIs)	• WizMonitor • Continuity of care workers • Internal HIS • WizMonitor • Tally sheets/Internal HIS • Internal HIS

^a^WizMonitor: Name of the data-collection software used by UNFPA

^b^Continuity of care workers: Staff that have been hired to collect and insert patient data on a daily basis. At the time of the assessment data was being inserted and stored in an excel sheet, however, IMC was in the process of developing an internal HIS system from the bottom-up

^c^Internal HIS: International Rescue Commission, UNRWA and UNHCR have implemented and utilize an internal HIS system for all their data collection and reporting efforts

### Ethical considerations

The findings from the KIIs, FGDs and facility assessments suggested that there are inconsistent and fragmented efforts for data protection and confidentiality surrounding SRMNCAH data and research across the different humanitarian agencies in Jordan. Health care facilities have basic protocols in place for data logging and protection, especially surrounding GBV data and access to the gender-based violence information management system (GBVMIS). However, the availability of data privacy and confidentiality protocols was directly correlated with the resources allocated for data collection and protection. Many agencies were able to run health care clinics and allocated funding to specialized Monitoring, Evaluation, Accountability and Learning (MEAL) departments and staff, institutional protocols, and a comprehensive HIS; yet, the majority of the health facilities we visited (n = 4) utilized Microsoft Excel for delivering and storing data. This was done mainly on an institution’s computer with a fixed password, an e-mail, a USB flash memory, or even tally-sheet printouts or the use of mobile phones. Some of the facilities visited kept a manual patient logbook could be accessed by ‘any’ staff member (n = 2), while the remaining facilities (n = 3) had specific (often the head nurse or midwife) on shift who had access to the master patient logbook.

All of our stakeholders discussed concerns with confidentiality and the national mandatory reporting requirements for GBV and early marriage. Participants explained that humanitarian organizations working in Jordan are mandated to abide by the country’s laws and regulations. This further exacerbates the underreporting of SGBV and early marriage cases for refugees residing in Jordan [26]. Similar challenges were noted surrounding the reporting of HIV and PMTCT-related indicators. Given that refugees face deportation if they are diagnosed with HIV [27], stakeholders had deep concerns about confidentiality, and most do not keep internal records. Noting that all refugees testing positive for HIV can only receive treatment through the MOH mandate and dedicated facility. As a participant explained,

We inform them [the HIV positive refugee] that we will have what we call a gentleman’s agreement with the MOH. When it comes to that refugee, if he or she found to be positive, we inform them that we will find a durable solution for the case, and that’s resettlement. So, they wait for us, until we get the settlement^3^ for the case.

## Discussion

**Fig. 3:**
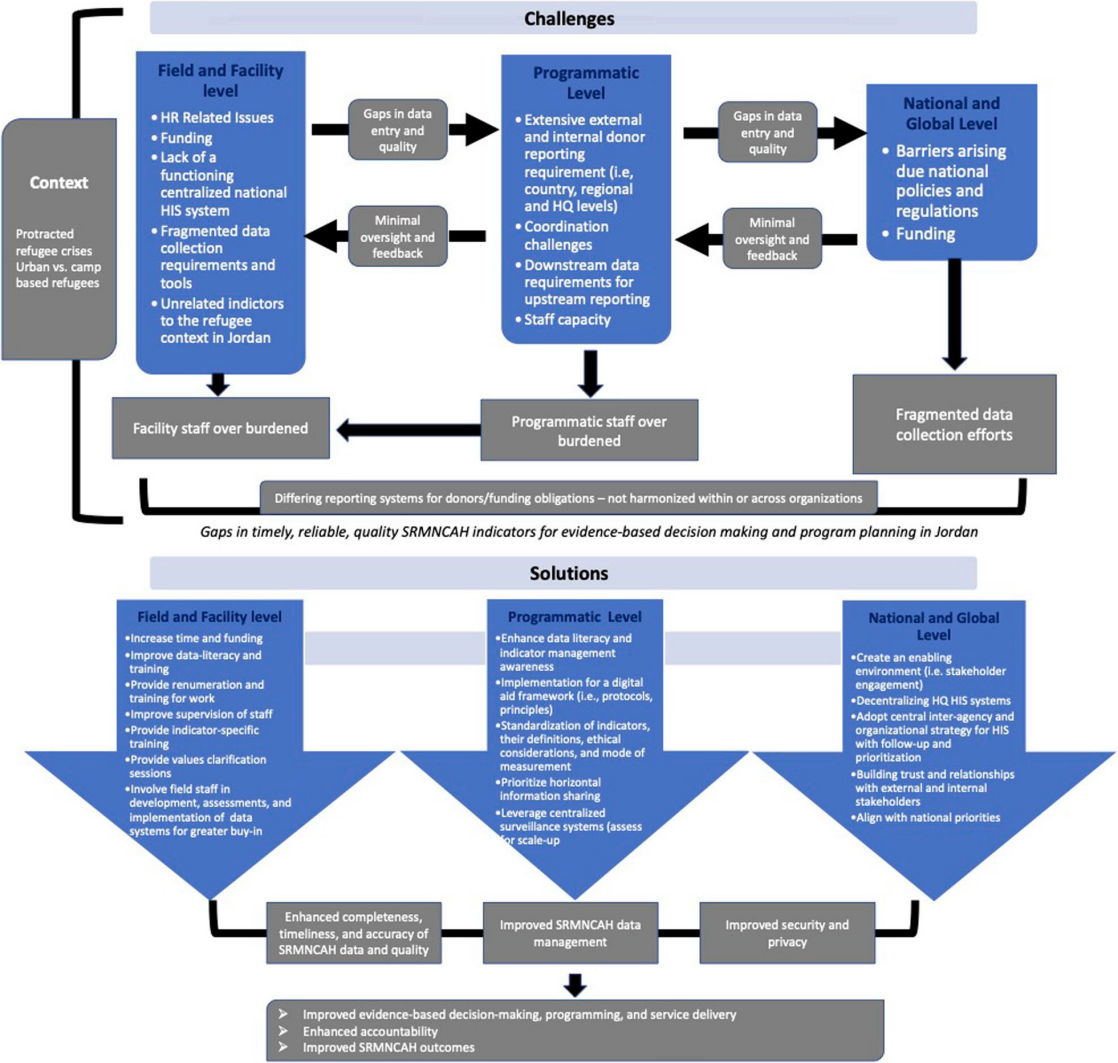
Challenges and solutions for timely, reliable, quality SRMNCAH indicator reporting at the facility, programmatic, and national and global levels in Jordan, reported by the study participants

The findings from this multi-methods feasibility assessment provide a comprehensive overview of the feasibility of collecting a core set of SRMNCAH indicators in the humanitarian context of Jordan for improved humanitarian response. The outlined recommendations are steppingstones for SRMNCAH service and outcomes monitoring and evaluation in humanitarian settings. The recommendations also aid communities, donors, and humanitarian actors in creating an enabling environment for quality SRMNCAH data collection and evidence-based decision making. In Fig. 3, we outline the policy, strategy, capacity, training, HR, and communication factors that are supported by the literature that need to be implemented and addressed at the global and national, programmatic and facility levels to increase the feasibility of current indicator reporting practices and the quality of SRMNCAH data reporting [12, 14, 28–31].

With a three-tiered health care system,^4^ limited budgets and varying policies and regulations in Jordan [32], the development of national priorities and guidelines for improving SRMNCAH indicators’ reporting for its population—and for refugee populations more specifically—has proven to be challenging. The literature indicates that despite the surfeit of data requested by the overarching humanitarian structure, researchers have found systematic reporting gaps for different SRMNCAH programming and evidence for impact on SRMNCAH and outcomes [15, 33, 34]. In 2014, Obrégon and colleagues concluded that there were major gaps in basic information needed to assess the quality and context of the data and reports they reviewed were lacking, irrespective of state of emergency or region [33]. A noted challenge to this by the research respondents, is that the requested data collection from donors, the national government, international and United Nations (UN) agencies, coordination/cluster systems is highly variable and burdensome. A mixed-methods assessment of routine health information systems and data conducted in Addis Ababa found that the extensive presence of parallel reporting and unstandardized routine data collection practices resulted in over and under reporting of health indicators and had negative implications on service provider’s perceptions toward routine health data collection practice; impacting data completeness and quality [35]. Jordanian stakeholders were clear that this core set of indicators would only be useful with buy-in from the global community that results in harmonizing and coordinating data collection efforts and relevant indicators’ reporting requirements. The literature outlines the incompatibility between de-centralized humanitarian organizational structure (i.e., allowing for a high degree of independence for sub and field offices, with minimal oversight by the headquarter (HQ), including HQ’s HIS infrastructure [16]. The availability of a robust computerized system will therefore directly depend on the budget and prioritization of monitoring systems [36], combined with a focus om preventing standardized horizontal communication and encouraging coordination efforts between and within different agencies [16]. Therefore, the sustainability of these data collection systems is directly dependent on the agencies’ and donors’ willingness to provide regular and continuous multi-year funding for national and international organizations, which currently is often crippled.

Stakeholders were clear that collecting the SRMNCAH indicators that required a population-level denominator may not be often feasible, particularly in urban environments. More than 70% of all Syrian refugees in Jordan reside outside of camps [1]. Although, these populations are concentrated in some areas, this population was only fully counted after completion of the most recent Jordan Population and Family and Health Survey (JPFHS), the results of which were published in 2018 [37]. Participants noted that the Demographic and Health Survey Program cycle is pre-determined. It requires significant resources and should continue to be collected and coordinated at the national level, which could impact the data quality of those indicators that rely on population level denominators. Study participants repeatedly emphasized their preference for a core set of facility-based indicators, as they perceived them to be more programmatically relevant and actionable. Guha-Sapir and Scales (2020) found that the use of facility-based data, preferably in the form of patient records, improved data analysis and quality, and strengthens the estimation of mortality and morbidity in emergency settings [38]. The study findings also suggest that facility-based indicators could be used as proxies to reflect recognized services delivery standards of SRMNCAH care in humanitarian settings, as most are pertinent to the Minimum Initial Services Package (MISP). Hence, for those indicators, even if they were not currently being collected, KIIs and FGD participants could generally envision how to incorporate this information into the facility-level data collection systems.

Our findings also indicated that there are socio-political as well as cultural barriers that could impede the collection of certain SRMNCAH indicators, specifically those surrounding sexual and gender-based violence (SGBV), abortion, and HIV/AIDs domains of indicators. A study exploring barriers of reporting of sexual violence in Al-Za’atari camp found that Syrian women did not report these cases, due to shame, limited trust in helpers and insufficient legal protections [26]. The literature shows that mobilizing innovative and validated methods such as “the Neighbourhood method” for estimating prevalence rates with an accuracy could be a suitable alternative for programmatic purposes in conflict-affected and post-conflict settings [39]. Adopting novel approaches for estimating abortion incidence could also help mitigate the socio-cultural barriers to access, disclosure and reporting [40]. Further, although Jordan does not currently have a dedicated product for emergency contraception, other modalities that can be used post-coitally including the use of the copper IUD and Yuzpe method are available [41]. Policies and legislative changes surrounding lack of approvals for a dedicated progestin-only EC should be considered, as this sends a signal that the medication is not safe or effective.

Stakeholders raised concerns about the inclusion of all the HIV/AIDS-related indicators due to major stigma and discrimination from service providers, as national reporting and treatment protocols preclude organizations from collecting data on HIV and prevention of mother-to-child transmission (PMTCT) [27, 42]. According to the Jordanian legislative system, medical professionals and health facilities are mandated to report an individual’s HIV status to the government [27]; foreign nationals, including refugees, who are found to be HIV-positive, are deported regardless of the consequences to their health and safety, and are not permitted to return to Jordan [42]. This highlights the need to reflect on the ethical implications; while considering the inclusion of these indicators. As, requiring reporting of HIV data without stringent protocols for data protection will only serve to increase stigma, which has been shown to delay enrolment in care for people living with HIV [27].

Given the need for consistency and accountability for monitoring and evaluation of SRMNACH services and outcomes in humanitarian settings, WHO, in collaboration with partner agencies, has supported the development of a ‘Data and Accountability Roadmap for Improving Data, Monitoring and Accountability for SRH in Crises’ [30]. This Roadmap responded to the need for collecting, aggregating, making accessible, using clear and consistent data; aligning with the SDGs, the ‘Every Women Every Child Strategy’, and the commitments made at the 2017 Family Planning Summit to address data gaps.

The findings from our assessment also indicated the need for resources, training, and capacity building to enhance the feasibility of accurate and ethical reporting on the proposed list of SRMNCAH indicators. An array of trainings accompanied with a tool kit guide on data collection for each of the SRMNCAH indicators’ domains is necessary before the rollout of the proposed indicators. The Jordanian context requires a centralized Health Information System that is harmonized and adequately coordinated with global and national reporting requirements to prevent overburdening staff at the data-collection level [29]. The system should be developed and tailored for use in the local context [10] as in the past, many data collection systems that were specifically designed for humanitarian actors were actually utilized in national systems with field workers. There should be a sustainable funded effort at the facility-level to update data collection systems and adapt medical records and logbooks, in order to create standardized data collection fields for the information derived from patient medical records. There must be capacity building at government-run organizations and local clinics to clean, correct, and annotate data.

## Strengths and limitations

The strategies recommended by Guba [43] were used for evaluating the trustworthiness of the data in this assessment. Credibility, confirmability, and transferability were used to ensure the trustworthiness of the data. Prolonged engagement, triangulation, member checks, peer-debriefing with the study team in both Jordan and Canada were used to ensure the credibility of the data [44]. The mixed-methods design of this study enabled the integration of data from multiple sources. Qualitative data was used to explore and explain the quantitative findings, with the findings from the facility assessments also validating (or in some cases dispelling) key themes identified in analyses of FGDs and KIIs. The facility assessments provided important findings relating to current management SRMNCAH monitoring and evaluation systems as well as the availability and distribution of specific resources. Despite these strengths, however, our study was limited by challenges with documentation, for example, despite extensive efforts, it was not always possible to locate and access SRMNCAH monitoring and evaluation records. The positionalities of the research team members undoubtedly influenced the participant-researcher interaction as well as our interpretation of data collected. Through memoing and regular debriefings, we attempted to reflect on and understand these dynamics, thereby enhancing the credibility and trustworthiness of the findings.

## Conclusion

The findings from our multi-methods feasibility assessment suggest that, overall, there is widespread support for developing a core and concise list of SRMNCAH indicators among humanitarian stakeholders in Jordan. Representatives from a variety of institutions noted numerous already existent resources and systems that could be leveraged, built upon, and improved to ensure the feasibility of collecting this core set of indicators for monitoring and SRMNCAH services and outcomes in humanitarian settings in Jordan. However, an important challenge to this was noted by the research respondents, which was that the requested data collection from donors, the national government, international and UN agencies, coordination/cluster systems is highly variable and burdensome. Stakeholders appreciated that a core set of SRMNCAH indicators would need to be globally relevant and feasible. However, they also clarified that need of this global set to be adequately aligned with national priorities in order to solicit a greater buy-in. The recent implementation of the Maternal Mortality Surveillance and Response System (MMSRS) instilled confidence among our participants that it would be possible to develop national, harmonized systems for reporting and aggregating information, provided that sufficient resources in both personnel and information technology is made available to build capacity and infrastructure.

Stakeholders were also clear that collecting indicators that required a population-level denominator in most instances is not feasible, particularly in urban environments. Health workers repeatedly emphasized their preference for a core set of facility-based indicators, as they perceived them to be more programmatically relevant and actionable. In addition to facility-based indicators, participants discussed the need for certain policy and regulatory changes in Jordan must be made before adopting the proposed indicators for programmatic relevance and actionability.

Finally, the Data and Accountability Roadmap for Improving Data, Monitoring and Accountability for SRH in Crises’ [30] combined with data literacy and indicator management awareness, digital aid frameworks and amendments of laws and regulations should be prioritized at the national and programmatic level to create an enabling environment to reduce duplicity, and improve communication channels and overarching SRMNCAH data quality [45].

Finally, it worth to reiterate that this assessment in Jordan is part of an extensive collaborative global effort that was culminated in June 2021, which lead to the finalization of the proposed M and E framework for SRMNCAH services and outcomes in humanitarian settings. During this global endorsement, and in line with the findings of this assessment, it was emphasized that it is crucial to consider the feasibility (logistical, socio-cultural and political), usefulness, ethical implications, and opportunity costs during the measurement and data collection of these SRMNCAH indicators, especially those indicators that are most sensitive.

## Data Availability

Available upon request.

## Abbreviations

BEmOC: Basic emergency obstetrical care
CEmOC: Comprehensive emergency obstetrical care
FGD: Focus Group Discussions
HIS: Health Information System
GBVMIS: Gender-based violence information management system
HIV/AIDS: Human Immunodeficiency Virus/ Acquired immunodeficiency syndrome
IAFM: Inter-Agency Field Manual on Reproductive Health
KI: Key Informant
KII: Key Informant Interview
KMC: Kangaroo mother care
MEAL: Monitoring, Evaluation, Accountability and Learning
MISP: Minimum Initial Service Package
MoH: Ministry of Health
NGO: Non-governmental organization
NSRHFP: National Strategy for Reproductive Health and Family Planning
PAC/CAC: Post abortion care/Comprehensive abortion care
RP2: Research Project Review Panel
SGBV: Sexual and gender-based violence
SRH: Sexual and reproductive health
SRMNCAH: Sexual, reproductive, maternal, neonatal, child, and adolescent health
UN: United Nations
UNFPA: United Nations Population Fund
UNHCR: United Nations High Commissioner for Refugees
UNICEF: United Nations Children's Fund
VCAT: Values clarification and attitudes transformation
WHO: World Health Organization

## Footnotes

1 A more detailed description of this projects’ methodology can be found in the report for the WHO.

2 LIVES Model developed by the World Health Organization: Includes a first-line support that involves five simple tasks; 1) **Listen** to the woman closely, with empathy, and without judging; 2) **Inquire about needs and concerns**, assess and respond to her various needs and concerns— emotional, physical, social and practical (eg.childcare); 3) **Validate**, show her that you understand and believe her. Assure her that she is not to blame; 4) **Enhance Safety,** discuss a plan to protect herself from further harm if violence occurs again; and 5) **Support** her by helping her connect to information, services and social support (47).

3 UNHCR will work to resettle any refugee who is HIV positive outside of Jordan to receive HIV-AIDS related services and treatment.

4 Health care services in Jordan are provided through the public/semipublic and private sector, and through Jordan’s Royal Medical Services (RMS). Services are provided through hospitals, primary care clinics, pharmacies and other ancillary services. In addition to the sectors, a significant portion of health services are provided through programs led by the United Nations and other international and national non-governmental humanitarian agencies.
